# Resurgent vector-borne diseases as a global health problem.

**DOI:** 10.3201/eid0403.980326

**Published:** 1998

**Authors:** D J Gubler

**Affiliations:** Division of Vector-Borne Infectious Diseases, National Center for Infectious Diseases, Centers for Disease Control and Prevention, Fort Collins, CO 80522, USA. djg2@cdc.gov

## Abstract

Vector-borne infectious diseases are emerging or resurging as a result of changes in public health policy, insecticide and drug resistance, shift in emphasis from prevention to emergency response, demographic and societal changes, and genetic changes in pathogens. Effective prevention strategies can reverse this trend. Research on vaccines, environmentally safe insecticides, alternative approaches to vector control, and training programs for health-care workers are needed.


### Resurgent Vector-Borne Diseases as a Global Health Problem

**Duane J. Gubler**
Centers for Disease Control and Prevention, Fort Collins, Colorado, USA

Vector-borne infectious diseases are emerging or resurging as a result of changes in public health policy, insecticide and drug resistance, shift in emphasis from prevention to emergency response, demographic and societal changes, and genetic changes in pathogens. Effective prevention strategies can reverse this trend. Research on vaccines, environmentally safe insecticides, alternative approaches to vector control, and training programs for health-care workers are needed.

In the 120 years since arthropods were shown to transmit human disease, hundreds of viruses, bacteria, protozoa, and helminths have been found to require a hematophagous (blood-sucking) arthropod for transmission between vertebrate hosts (1). Historically, malaria, dengue, yellow fever, plague, filariasis, louse-borne typhus, trypanosomiasis, leishmaniasis, and other vector-borne diseases were responsible for more human disease and death in the 17th through the early 20th centuries than all other causes combined (1). During the 19th and 20th centuries, vector-borne diseases prevented the development of large areas of the tropics, especially in Africa; it was not until these diseases were controlled that engineering feats such as the Panama Canal could be completed (1,2).

Not long after the 1877 discovery that mosquitoes transmitted filariasis from human to human, malaria (1898), yellow fever (1900), and dengue (1903) were shown to have similar transmission cycles (2). By 1910, other major vector-borne diseases such as African sleeping sickness, plague, Rocky Mountain spotted fever, relapsing fever, Chagas disease, sandfly fever, and louse-borne typhus had all been shown to require a blood-sucking arthropod vector for transmission to humans (2).

Prevention and control programs were soon based on controlling the arthropod vector. Yellow fever in Cuba was the first vector-borne disease to be effectively controlled in this manner, followed quickly by yellow fever and malaria in Panama. Over the next 50 years, most of the important vector-borne public health problems were effectively controlled (Table 1). Most of these programs established vertically structured vector control organizations that emphasized elimination of arthropod breeding sites (source reduction) through environmental hygiene along with limited use of chemical insecticides. By the 1960s, vector-borne diseases were no longer considered major public health problems outside Africa. Urban yellow fever and dengue, both

Table 1. Successful vector-borne disease control/elimination programs

| Disease | Location | Year(s) |
|---|---|---|
| Yellow fever | Cuba | 1900-1901 |
| Yellow fever | Panama | 1904 |
| Yellow fever | Brazil | 1932 |
| *Anopheles gambiae* infestation | Brazil | 1938 |
| *An. gambiae* infestation | Egypt | 1942 |
| Louse-borne typhus | Italy | 1942 |
| Malaria | Sardinia | 1946 |
| Yellow fever (*Aedes aegypti*) | Americas | 1947-1970 |
| Malaria | Americas | 1954-1975 |
| Malaria | Global | 1955-1975 |
| Yellow fever | West Africa | 1950-1970 |
| Onchocerciasis | West Africa | 1974-present |
| Bancroftian filariasis | South Pacific | 1970s |
| Chagas disease | South America | 1991-present |

Address for correspondence: Duane J. Gubler, Division of Vector-Borne Infectious Diseases, National Center for Infectious Diseases, Centers for Disease Control and Prevention, P.O. Box 2087, Fort Collins, CO 80522, USA; fax: 970-221-6476; e-mail: djg2@cdc.gov.

transmitted by *Aedes aegypti*, were effectively controlled in Central and South America and eliminated from North America; malaria was nearly eradicated in the Americas, the Pacific Islands, and Asia. The discovery and effective use of residual insecticides in the 1940s, 1950s, and 1960s contributed greatly to these successes.

However, the benefits of vector-borne disease control programs were short-lived. A number of vector-borne diseases began to reemerge in the 1970s, a resurgence that has greatly intensified in the past 20 years (3-7). Although the reasons for the failure of these programs are complex and not well understood, two factors played important roles: 1) the diversion of financial support and subsequent loss of public health infrastructure and 2) reliance on quick-fix solutions such as insecticides and drugs.

### The Global Emergence/Resurgence of Vector-Borne Diseases

Evidence of the reemergence of vector-borne diseases such as malaria and dengue was first observed in the 1970s in Asia and the Americas (5-9). Warnings, however, were largely ignored until recently (10), and now it may be difficult to reverse the trend.

Figure 1 shows some vector-borne parasitic, bacterial, and viral diseases that have caused epidemics in the 1990s. While malaria is the most important vector-borne disease because of its global distribution, the numbers of people affected, and the large number of deaths, the vector-borne viruses (arboviruses) are clearly the most numerous.

### Malaria

The resurgence of malaria in Asia in the late 1960s and early 1970s provides a dramatic example of how quickly vector-borne disease trends can change. Malaria, transmitted to humans by anopheline mosquitoes, had been nearly eliminated in Sri Lanka in the 1960s, with only 31 and 17 cases reported in 1962 and 1963, respectively. By 1967, 3,468 cases were reported. In 1968, however, a major epidemic caused 440,644 cases. In 1969, 537,705 cases were reported (Figure 2a); the disease has never been effectively controlled since then. In India, a similar resurgence of malaria occurred (Figure 2b), with sporadic outbreaks of disease beginning in the early 1970s and nearly seven million cases by 1976. Sri Lanka and India are classic examples of the lack of sustainability of vertically structured prevention/control/elimination programs. Complacency, dwindling financial and political support, and a change in strategy from vector control to case finding and drug treatment were mainly responsible for the resurgence of malaria in these countries.

More recently, vivax malaria has reemerged in Korea (Figure 2c). Urban malaria in the Indian subcontinent and in parts of South America (Figure 2d) is also a major concern. In 1998, malaria is the most important tropical disease with more than half of the world's population living in areas of risk and with an estimated 200 million cases and two million deaths each year (11). Widespread drug resistance of the parasites and insecticide resistance among anopheline mosquito vectors have complicated malaria control (4).

Malaria is the most common imported disease in the United States, where anopheline mosquito vectors still exist (12). Approximately 1,000 suspected malaria cases are imported into the United States each year, associated with increased frequency of autochthonous cases; since 1987, 16 incidents of autochthonous malaria have occurred in nearly all parts of the United States. In each incident, however, transmission was limited to only a few cases (12).

### African Trypanosomiasis

Historically, African sleeping sickness, transmitted by the tsetse fly, has been a major impediment to the social and economic development of Central and East Africa. With the use of modern drugs, insecticides, and other control methods, this disease was effectively controlled in most countries by the mid-1960s. In the past 20 years, however, major epidemics have occurred in East and Central Africa, mainly because control programs were disrupted by war (13). In the Sudan, the Republic of Congo, and Angola, which have high prevalence, poor surveillance, no drugs, and no vector control, the disease poses a major public health threat. Although available, some new drugs, vector control approaches, and diagnostic tests are not being used because of lack of funding support.

African sleeping sickness is a low-priority rural disease. Effective, sustainable control is unlikely until traditional uses of land change and socioeconomic conditions improve in rural Africa (13). The primary approach to control is treatment with drugs that are expensive and not

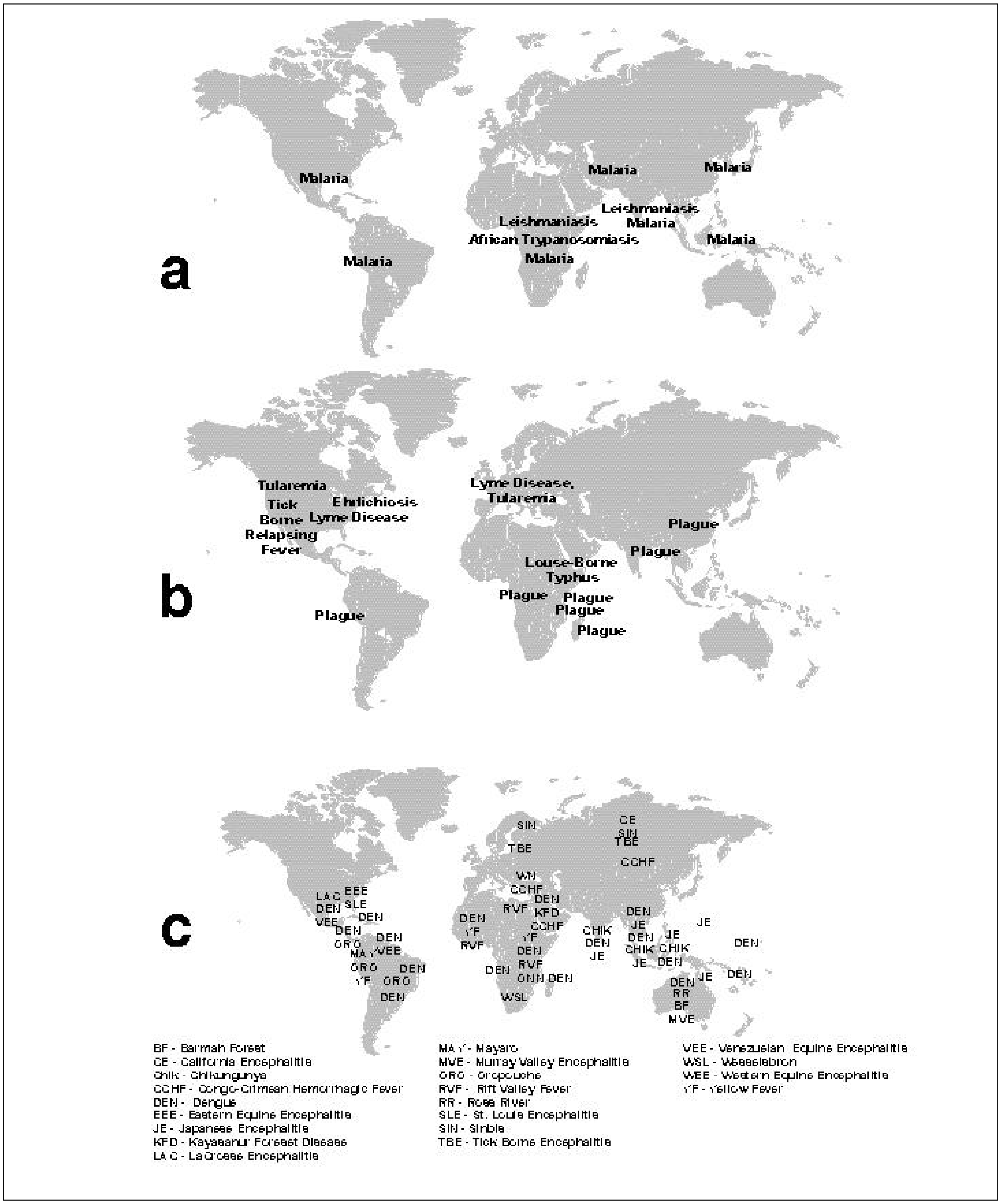

Figure 1. Epidemic vector-borne diseases, 1990–1997. A. parasitic diseases, B. bacterial diseases, C. arboviral diseases.

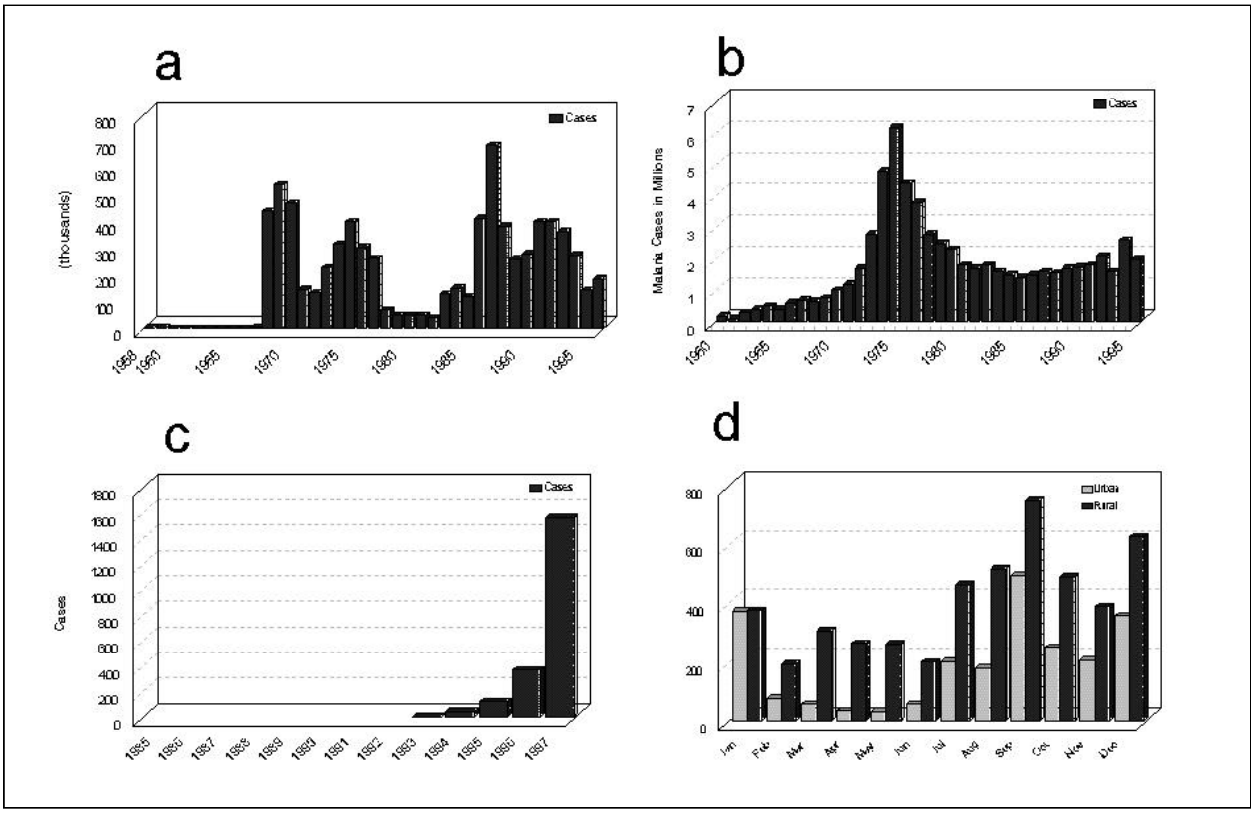

Figure 2. The resurgence of malaria. A. Sri Lanka (data from Tissa Vitarana, Office of Science and Technology, Sri Lanka); B. India (data from Shiv Lal, Director, National Malaria Eradication Program, India); C. Korea (data from Dan Strickman, Walter Reed Army Institute of Research; D. Manaus, Brazil (data from Bedsy Dutary, National Institute of Research of the Amazon).

readily available (11). To reverse this trend, an integrated sustainable control program must be implemented, including effective surveillance for case finding, a network of treatment centers with a supply of drugs, and vector control using trapping techniques (13).

### Lyme Disease

Lyme disease, a bacterial tick-borne infection, is caused by *Borrelia burgdorferi*. Discovered in the United States in 1975, the disease has continued to increase in incidence and geographic distribution since national surveillance was initiated in 1982. At that time, 497 cases were reported compared with 11,700 to 16,455 cases each year between 1994 and 1997 (Figure 3) (cumulative total cases reported more than 109,000) (14). Lyme disease has a global distribution in the temperate regions. Because of the multistage disease and chronic complications associated with *B. burgdorferi* infection, Lyme disease has major public health and economic effects.

Lyme disease is transmitted by *Ixodes ricinus* complex hard ticks. In the United States, *I. scapularis*, the deer tick, is the vector in the eastern and midwestern states, and *I. pacificus* is the vector in the far western states. While human cases of apparent Lyme disease are reported from most states and many enzootic cycles of *B. burgdorferi* occur throughout the country, the public health importance of these cases is uncertain. Approximately 90% of reported Lyme disease cases occur each year in the Northeast (Connecticut, Maryland, Massachusetts, New Jersey, New York, Pennsylvania, and Rhode Island), upper Midwest (Minnesota and Wisconsin), and Northwest (California) (14).

### Plague

Plague is the original emerging disease, having caused major pandemics; the most recent (late 19th century) is believed to be responsible for the current global distribution of the disease, which is spread by rats on ships (15). Like many other

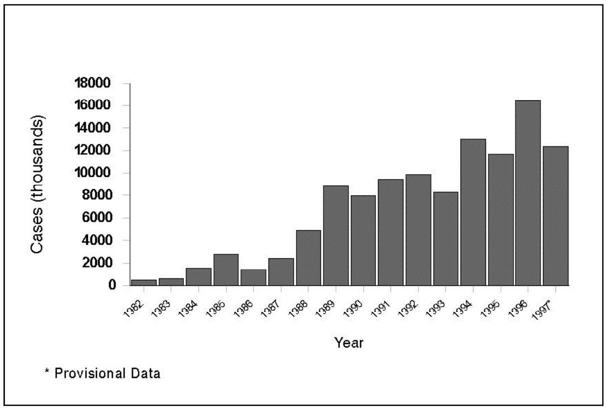

Figure 3. Reported cases of Lyme disease in the United States, 1982–1997.

vector-borne diseases, plague was controlled with antibiotics, insecticides, and rat control in the latter half of the 20th century. The number of cases reported to the World Health Organization decreased to an all-time low of 200 cases in 1981 (15). In recent years, however, epidemic plague has resurged, most notably in Africa, with an average of nearly 3,000 cases reported annually (approximately 65% from Africa) (15).

The decrease in plague incidence from 1950 to 1980 was followed by decreased financial support, lowered interest, and ultimately the deterioration of surveillance systems. Many countries were no longer capable of making a laboratory diagnosis of plague in the 1990s. For example, in 1994 when an outbreak of plague occurred in Western India (16) (which had reported its last case of plague in 1966), lack of laboratory capacity for diagnosis led to confusion as to the cause of the outbreak and panic within the population. An estimated 500,000 people fled Surat for other major cities, some of which subsequently reported secondary plague transmission (16).

Because effective epidemiologic investigation and an accurate laboratory diagnosis were not made in time, a relatively unimportant, focal public health event turned into an international public health emergency costing the Indian and the global economies billions of U.S. dollars (17). Plague can cause explosive epidemics when effective laboratory-based surveillance and prevention and control are not maintained in countries with enzootic disease. An important lesson learned from this incident was that laboratory-based international infectious disease surveillance is cost-effective.

### Dengue

Dengue fever caused major epidemics from the 17th to the early 20th centuries (18). In most Central and South American countries, effective disease prevention was achieved by eliminating the principal epidemic mosquito vector, *A. aegypti,* during the 1950s and 1960s. In Asia, however, effective mosquito control was never achieved, and a severe hemorrhagic fever (DHF) emerged following World War II. During the 1950s, 1960s, and 1970s, this new form of dengue occurred as periodic epidemics in a few countries. During the 1980s, however, incidence increased dramatically, expanding distribution of the virus and the mosquito vector to the Pacific islands and tropical America (18). In the latter region, the *Ae. aegypti* eradication program had been disbanded in the early 1970s; by the 1980s, this species had reinfested most tropical countries of the region (Figure 4). Increased disease transmission and frequency of epidemics caused by multiple virus serotypes in Asia increased the movement of dengue viruses into these regions, resulting in a dramatic increase in epidemic dengue fever; hyperendemicity (the cocirculation of multiple virus serotypes); and the emergence of DHF in the Pacific Islands, the Caribbean, and Central and South America. Thus, in less than 20 years, both the American tropics and the Pacific Islands went from not having dengue to having an important dengue/DHF problem in 1998.

Globally, DHF has emerged as a major cause of hospitalization and death. The number of DHF cases reported from 1981 to 1995 is four times higher than that of the previous 30 years. In 1998, more than 2.5 billion persons live in areas of risk (Figure 5). Dengue is the second most important tropical disease (after malaria) with approximately 50 to 100 million cases of dengue fever and 500,000 cases of DHF each year.

Because of limited surveillance data and gross underreporting in most disease-endemic countries, the economic and public health impact of dengue is greatly underestimated.

### Yellow Fever

Like dengue fever, yellow fever caused major epidemics from the 17th to the 20th centuries and was effectively controlled in the Americas by the *Ae. aegypti* elimination program in the 1950s and 1960s. Yellow fever is maintained in forest cycles involving monkeys and canopy-dwelling mosquitoes in both Africa and the Americas.

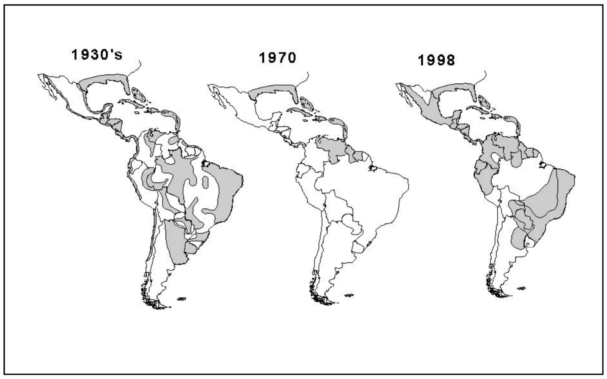

Figure 4. Geographic distribution of *Aedes aegypti* in the Americas, 1930s, 1970, and 1998.

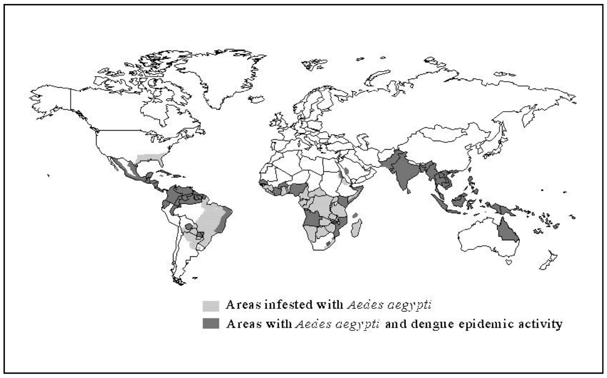

Figure 5. Global distribution of *Aedes aegypti* and of epidemic dengue, 1980–1998.

Human infections since the 1950s have been primarily in persons associated with the forest. Since the mid-1980s, however, epidemic yellow fever has resurged in West Africa, and for the first time in history, an outbreak occurred in Kenya in 1992 to 1993 (19).

Although the last urban epidemic in the Americas was in 1942 (20), urban epidemics may recur because nearly all major urban centers of the American tropics have been reinfested by *Ae. aegypti* in the past 20 years. Most persons in tropical American cities are at high risk for epidemic urban transmission because of low yellow fever immunity. Of added concern are the increasingly frequent reports of imported yellow fever to mosquito-infested urban areas (Figure 6). In the past 2 years, yellow fever cases have been imported to Santa Cruz, Bolivia; Manaus, Brazil; Villavicencia, Colombia; and Iquitos, Peru, all urban centers infested with *Ae. aegypti*. Moreover, two patients with yellow fever cases imported to the United States and Switzerland died; neither patient had been vaccinated.

Thus, the frequency of yellow fever moving from the American rain forest to tropical urban areas is increasing, and it is likely only a matter of time before an urban yellow fever epidemic will occur. The disease will then likely spread rapidly to other cities in the Americas and from there to cities in Asia and the Pacific, much as dengue has in the past 20 years (18). Because of the similarities in clinical expression between yellow fever and other common diseases such as dengue and leptospirosis and because the surveillance systems needed to detect yellow fever are very limited in most countries, widespread epidemic transmission would likely occur before the disease is detected. Emergency methods of controlling *Ae. aegypti* are ineffective (21,22); therefore, a major international public health emergency could occur.

These are only a few examples of emergent/resurgent vector-borne diseases, but there are many more that are causing increasingly frequent epidemics. Many go unreported because laboratory-based surveillance systems are not available in many countries.

### Factors Involved in Vector-Borne Disease Emergence

The factors responsible for the emergence/resurgence of vector-borne diseases are complex. They include insecticide and drug resistance, changes in public health policy, emphasis on emergency response, deemphasis of prevention programs, demographic and societal changes,

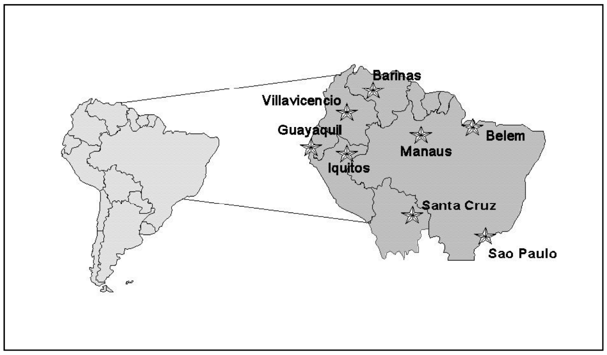

Figure 6. Major urban centers of South America recently infested with *Aedes aegypti* and at high risk for imported yellow fever.

and genetic changes in pathogens (10). Public health policy decisions have greatly decreased the resources for surveillance, prevention, and control of vector-borne diseases in the 1960s and 1970s, primarily because control programs had reduced the public health threat from these diseases. Those decisions, the technical problems of insecticide and drug resistance, as well as too much emphasis on insecticide sprays to kill adult mosquitoes, contributed greatly to the resurgence of diseases such as malaria and dengue. Decreased resources for infectious diseases in general resulted in the discontinuation or merger of many programs and ultimately to the deterioration of the public health infrastructure required to deal with these diseases. Moreover, good training programs in vector-borne diseases decreased dramatically after 1970. Thus, in 1998, we are faced with a critical shortage of specialists trained to respond effectively to the resurgence of vector-borne diseases (10,23). A related problem is the lack of preventive medicine training in most medical schools. The curative approach and emphasis on high-tech solutions to disease control have led most physicians, health officials, and the public to rely on "magic bullets" to cure an illness or control an epidemic (21).

Major global demographic and societal changes of the past 50 years have directly affected the emergence/resurgence of vector-borne and other infectious diseases (10,21,23,24). Unprecedented population growth, mostly in developing countries, resulted in major movements of people, primarily to urban centers. This unplanned and uncontrolled urbanization (inadequate housing, deteriorating water, sewage, and waste management systems) produced ideal conditions for increased transmission of mosquito-borne, rodent-borne, and water-borne diseases. The prospects for the future are not good; nearly all of the world's population growth in the next 25 years will occur in the urban centers of developing countries, many of them in tropical areas where vector-borne diseases occur most frequently (25).

Other societal changes, such as agricultural practices and deforestation (10), increase the risk for vector-borne disease transmission (Table 2). Many irrigation systems and dams have been built in the past 50 years without regard to their effect on vector-borne diseases. Similarly, tropical forests are being cleared at an increasing rate, and agricultural practices such as rice production have also increased.

Consumer products make ideal breeding sites for domesticated mosquitoes. Packaged in nonbiodegradable plastics, cellophanes, and tin, these products tend to be discarded in the environment where they collect rainwater. Discarded automobile tires, many in the domestic environment, make ideal mosquito breeding places as well as rat and rodent harborages. Container shipping and the global used tire industry have contributed to the increased geographic distribution of selected mosquito species that lay their eggs in used tires (26).

Finally, the jet airplane has had a major influence on global demographics (27). The airplane provides the ideal mechanism for transporting pathogens between population centers (10,18,21,23). The result is a constant movement of viruses, bacteria, and parasites among cities, countries, regions, and continents.

Climate change (e.g., global warming and El Niño Southern Oscillation) is often cited as the cause for the emergence/resurgence of vector-borne diseases, especially malaria, dengue, and

Table 2. Influences on emergent/resurgent vector-borne diseases

| Urbanization | Deforestation | Agricultural Practices |
|---|---|---|
| Dengue fever | Loaiasis | Malaria |
| Malaria | Onchocerciasis | Japanese encephalitis |
| Yellow fever | Malaria | St. Louis encephalitis |
| Chickungunya | Leishmaniasis | West Nile fever |
| Epidemic polyarthritis | Yellow fever | Oropouche |
| West Nile fever | Kyasanur Forest disease | Western equine encephalitis |
| St. Louis encephalitis | La Crosse encephalitis | Venezuelan equine encephalitis |
| Lyme disease | Eastern equine encephalitis | |
| Ehrlichiosis | Lyme disease | |
| Plague | | |

yellow fever. While meteorologic factors such as temperature, rainfall, and humidity influence the transmission dynamics of vector-borne diseases, climate change has not yet been scientifically proven to have caused the emergence/resurgence of any of the vector-borne diseases described above.

### The Future

Reversing the trend of emergent/resurgent vector-borne diseases is a major challenge. Vaccines, available for only a few diseases (yellow fever, Japanese encephalitis, tick-borne encephalitis, tularemia, plague), are not widely used. Vaccine prospects for major vector-borne diseases are not good. With the exception of malaria, few other vector-borne diseases have funding for vaccine research.

In the next decade, therefore, vector control will be required to interrupt transmission of most emergent/resurgent vector-borne diseases. Environmentally safe insecticides and research on alternative approaches (such as biological control) are needed. Integrated prevention strategies must be developed and implemented in endemic/enzootic-disease areas. In addition to economic support for research, human resources are needed to develop and implement sustainable prevention programs. Adequately trained personnel are lacking in most developing countries, as are academic institutions with the programs to train them. Policy changes must be made to support public health approaches to disease prevention. All these factors are needed to rebuild the public health infrastructure. Ultimately, however, demographic trends that have resulted in increased population pressure on urban centers and changes in agricultural practices must be reversed. Only then will we be able to effectively reverse the current trend of emergent/resurgent vector-borne disease in the 21st century.

### Acknowledgment

The author is grateful to numerous colleagues around the world for contributing data and information used in preparation of this paper.

Dr. Gubler is director of the Division of Vector-Borne Infectious Diseases, National Center for Infectious Diseases, CDC, in Fort Collins, Colorado. His research interests include field epidemiology, laboratory diagnosis, surveillance, prevention, and control of vector-borne diseases, with special emphasis on dengue/dengue hemorrhagic fever and other arboviruses.

### References

1. Gubler DJ. Insects in Disease Transmission. In: Strickland GT, editor. Hunter tropical medicine, 7th edition. Philadelphia (PA): W. B. Saunders; 1991. p. 981-1000.
2. Philip CB, Rozenboom LE. Medico-veterinary entomology: a generation of progress. In: Smith RF, Mittler TE, Smith CN, editors. History of entomology. Palo Alto (CA): Annual Reviews Inc; 1973.
3. Gubler DJ. The global resurgence of arboviral diseases. Trans Roy Soc Trop Med Hyg 1996;90:449-51.
4. Krogstad DJ. Malaria as a reemerging disease. Epidemiol Rev 1996;18:77-89.
5. Bruce-Chwatt LJ. The Manson Oration, May 1979. Man against malaria: conquest or defeat? Trans Roy Soc Trop Med Hyg 1979;73:605-17.
6. Hammon WM. Dengue hemorrhagic fever—do we know its cause? Am J Trop Med Hyg 1973;22:81-91.
7. Pan American Health Organization. Dengue in the Caribbean, 1977. Scientific Publication No. 375. Washington: The Organization; 1979.
8. Reeves WC. Recrudescence of arthropod-borne diseases in the Americas. Washington: Pan American Health Organization; 1972. PAHO Scientific Publication No. 238. DC.
9. Groot H. The reinvasion of Colombia by *Aedes aegypti*: aspects to remember. Am J Trop Med Hyg 1980;29:330-8.
10. Lederberg J, Shope RE, Oaks SC Jr, editors. Emerging infections: microbial threats to health in the United States. Washington: National Academy Press; 1992.
11. The World Health Report 1996: fighting disease, fostering development. Geneva: World Health Organization; 1996.
12. Zucker JR. Changing patterns of autochthonous malaria transmission in the United States: a review of recent outbreaks. Emerg Infect Dis 1006;2:37-43.
13. Molyneux DH. Current public health status of the trypanosomiases and leishmaniases. In: Hilde G, Mottram JC, Coombs GH, Holmes PH, editors. Trypanosomiasis and leishmaniasis. London: CAB International; 1997. p. 39-50.
14. Dennis DT. 1998. Epidemiology, ecology, and prevention of Lyme disease. In: Rahn DW, Evens J, editors. Lyme disease. Philadelphia (PA): American College of Physicians; 1998. p. 7-34.
15. Dennis DT. Plague as an emerging disease. Emerging Infections II. In press 1998.
16. Ramalingaswami V. The plague outbreaks of India, 1994–a prologue. Current Science 1996;71:781-806.
17. Plague—India 1994: economic loss. Geneva: World Health Organization; 1997. p. 14.
18. Gubler DJ. Dengue and dengue hemorrhagic fever: its history and resurgence as a global public health problem. In: Gubler DJ, Kuno G, editors. Dengue and dengue hemorrhagic fever. London: CAB International. p. 1-22.
19. Sanders EJ, Tukei PM. Yellow fever: an emerging threat for Kenya and other East African countries. East Afr Med J 1996;73:10-2.
20. Monath TP. Yellow fever. In: Monath TP, editor. The arboviruses: epidemiology and ecology. Boca Raton (FL): CRC Press; 1988. p. 139-231.

21. Gubler DJ. *Aedes aegypti* and *Aedes aegypti*-borne disease control in the 1990s: top down or bottom up. Am J Trop Med Hyg 1989;40:571-8.
22. Reiter P, Gubler DJ. Surveillance and control of urban dengue vectors. In: Gubler DJ, Kuno G, editors. Dengue and dengue hemorrhagic fever. London: CAB International; 1997. p. 425-62.
23. Gubler DJ. Epidemic dengue and dengue hemorrhagic fever: a global public health problem in the 21st century. In: Scheld WM, Armstrong D, Hughes JM editors. Emerging infections 1. Washington: ASM Press; 1997. p. 1-14.
24. Addressing emerging infectious disease threats: a prevention strategy for the United States. Atlanta (GA): Centers for Disease Control and Prevention; 1994.
25. World resources 1996-97. A guide to the global environment. The urban environment. New York: Oxford University Press: 1996.
26. Reiter P, Sprenger D. The used tire trade: a mechanism for the worldwide dispersal of container breeding mosquitoes. J Am Mosq Ctrl Assoc 1987;3:494-501.
27. Gubler DJ. Arboviruses as imported disease agents: the need to increased awareness. Arch Virol 1996;11:21-32.
